# Intensive weekly chemotherapy is not effective in advanced pancreatic cancer patients: a report from the Italian Group for the Study of Digestive Tract Cancer (GISCAD)

**DOI:** 10.1038/sj.bjc.6690076

**Published:** 1999-02

**Authors:** S Cascinu, L Frontini, G Comella, S Barni, R Labianca, N Battelli, R Casaretti, S Zonato, M Pirovano, G Catalano, R Cellerino

**Affiliations:** Departments and Units of Medical Oncology, Ancona, Naples, Milan, Monza, Pesaro, Italy

**Keywords:** pancreatic cancer, intensive chemotherapy, palliation

## Abstract

Twenty-two patients, with locally advanced unresectable and/or metastatic pancreatic carcinoma, received weekly administration of cisplatin 40 mg m^−2^, 5-fluorouracil 500 mg m^−2^, epidoxorubicin 35 mg m^−2^, 6S stereoisomer of leucovorin 250 mg m^−2^ and glutathione 1.5 mg m^−2^, supported by a daily administration of lenograstim at a dose of 5 μg kg^−1^. Nineteen patients were men and three were women. Median age was 63 years (range 47–70). At study entry, pain was present in 15 out of 22 patients (68%) with a mean value of Scott–Huskisson scale of 27.6 ± 23.8, whereas a weight loss >10% was present in 15 patients. After eight weekly treatments, three partial responses were achieved for a response rate of 13% (95% CI 0–26%), five patients had stable disease and 14 progressed on therapy. Pain was present in 9 out of 22 patients (40%) with a mean value of Scott–Huskisson scale of 12.3 ± 18.4. Eight patients (36%) (three partial response and five stable disease) had a positive weight change. Toxicity was mild: WHO grade III or IV toxicity was recorded in terms of anaemia in 7 out of 188 cycles (3.7%), of neutropenia in 9 out of 188 cycles (4.7%) and of thrombocytopenia in 3 out of 188 cycles (1.5%). Median survival of all patients was 6 months. The outcome of this intensive chemotherapy regimen does not support its use in pancreatic cancer. © 1999 Cancer Research Campaign


British Journal of Cancer (1999) **79**(3/4), 491–494
© 1999 Cancer Research Campaign
Article no. bjoc.1998.0076

### Intensive weekly chemotherapy is not effective in advanced pancreatic cancer patients: a report from the Italian Group for the Study of Digestive Tract Cancer (GISCAD)

**S Cascinu, L Frontini, G Comella, S Barni, R Labianca, N Battelli, R Casaretti, S Zonato, M Pirovano, G Catalano and R Cellerino**

Departments and Units of Medical Oncology, Ancona, Naples, Milan, Monza, Pesaro, Italy

**Summary** Twenty-two patients, with locally advanced unresectable and/or metastatic pancreatic carcinoma, received weekly administration of cisplatin 40 mg $m^{-2}$, 5-fluorouracil 500 mg $m^{-2}$, epidoxorubicin 35 mg $m^{-2}$, 6S stereoisomer of leucovorin 250 mg $m^{-2}$ and glutathione 1.5 mg $m^{-2}$, supported by a daily administration of lenograstim at a dose of 5 µg $kg^{-1}$. Nineteen patients were men and three were women. Median age was 63 years (range 47–70). At study entry, pain was present in 15 out of 22 patients (68%) with a mean value of Scott–Huskisson scale of 27.6 ± 23.8, whereas a weight loss >10% was present in 15 patients. After eight weekly treatments, three partial responses were achieved for a response rate of 13% (95% CI 0–26%), five patients had stable disease and 14 progressed on therapy. Pain was present in 9 out of 22 patients (40%) with a mean value of Scott–Huskisson scale of 12.3 ± 18.4. Eight patients (36%) (three partial response and five stable disease) had a positive weight change. Toxicity was mild: WHO grade III or IV toxicity was recorded in terms of anaemia in 7 out of 188 cycles (3.7%), of neutropenia in 9 out of 188 cycles (4.7%) and of thrombocytopenia in 3 out of 188 cycles (1.5%). Median survival of all patients was 6 months. The outcome of this intensive chemotherapy regimen does not support its use in pancreatic cancer.

**Keywords:** pancreatic cancer; intensive chemotherapy; palliation

Pancreatic cancer is a rapidly fatal disease, with a 5-year survival rate of less than 5% (Kelly and Benjamin, 1995). Surgery has been considered the only curative modality for this disease, even if at the time of diagnosis the majority of patients have locally advanced unresectable or metastatic disease (Casper and Kelsen, 1995). Until very recently, chemotherapy was held to be largely ineffective in terms of objective responses, survival or quality of life in advanced pancreatic cancer patients. Editorials and reviews called repetitively for abandonment of chemotherapy because no clear benefit was evident (Taylor, 1993; Lionetto et al, 1995). However, in the last 2 years, opinions about the value of chemotherapy in advanced pancreatic cancer have varied. Several trials have shown that chemotherapy can prolong survival and improve quality of life in advanced pancreatic cancer (Palmer et al, 1994; André et al, 1996; Glimelius et al, 1996; Rothenberg et al, 1996; Burris et al, 1997).

Recently, more aggressive chemotherapy regimens have been reported to be highly effective in advanced gastric cancer (Cocconi et al, 1994; Webb et al, 1997). In our multi-institutional trial, a weekly low dose of cisplatin (CDDP), epidoxorubicin (epi-ADR), leucovorin (LV) and 5-fluorouracil (5-FU) determined a high response rate (62%) and an interesting median survival (11.7 months) in 105 advanced gastric cancer patients (Cascinu et al, 1997). On the basis of these results, and of the favourable results obtained by chemotherapy in recent studies, we considered this weekly regimen worthy of evaluation in the management of patients with advanced carcinoma of the pancreas.

*Received 17 December 1997*
*Revised 24 March 1998*
*Accepted 15 June 1998*

*Correspondence to:* S Cascinu, Sezione di Oncologia Sperimentale, Unità Operativa di Oncologia Medica, Azienda Ospedaliera ‘Ospedale S. Salvatore’ Pesaro, Via Lombroso 61100 Pesaro, Italy

### PATIENTS AND METHODS

### Patient selection

Patients with histologically verified locally advanced unresectable and/or metastatic pancreatic carcinoma were eligible for the study. Patients thought to have potentially curable disease by resection of the primary were not eligible. Other eligibility criteria included performance status Eastern Cooperative Oncology Group grade 0–3, age less than 70 years, and normal liver (serum bilirubin <1.5 mg $dl^{-1}$), renal (serum creatinine <1.5 mg $dl^{-1}$) and bone marrow (leucocyte count >4000 µ$l^{-1}$, platelet count >100 000 µ$l^{-1}$) functions. Because epi-ADR was included in the treatment plan, patients had to have a New York Heart Association class of ≤2; if there was a history of cardiac disease, a cardiac-gated pool scan with an ejection fraction of >45% was required.

Patients were excluded if they had previously undergone chemotherapy. Patients who had had radiotherapy to individual sites of disease were eligible, but that (those) site(s) of disease was (were) considered non-evaluable for response.

Informed consent was obtained from all participants after the nature of the study had been fully explained, and the protocol was approved by the institutional review board.

**Table 1** Patient characteristics

| | |
|---|---|
| No. of patients | 22 |
| Age (years) | |
| Median | 63.5 |
| Range | 47–70 |
| Sex | |
| Men/Women | 19/3 |
| Performance status (ECOG) | |
| 0 | 2 |
| I | 10 |
| II | 9 |
| III | 1 |
| Disease at presentation | |
| Locally advanced | 4 |
| Metastatic | 16 |
| Locally advanced and metastatic | 2 |
| Site of primary tumour | |
| Head | 7 |
| Body | 8 |
| Tail | 7 |
| Histology | |
| Well differentiated | 15 |
| Moderately differentiated | 5 |
| Poorly differentiated | 2 |
| Symptoms | |
| Pain | 15 |
| Dyspepsia | 9 |
| Weight loss >10% | 15 |

### Chemotherapy

The chemotherapeutic regimen consisted of a 1-day weekly administration of CDDP 40 mg m$^{-2}$ as a 30-min infusion in 250 ml of normal saline solution, 5-FU 500 mg m$^{-2}$ as a 15-min infusion in 100 ml of normal saline solution, epi-ADR 35 mg m$^{-2}$ by intravenous bolus. 6S stereoisomer of leucovorin was administered at a dose of 250 mg m$^{-2}$ diluted in 250 ml of normal saline solution in a 4-h infusion concurrent with hydration.

Glutathione was given at a dose of 1.5 g m$^{-2}$ in 100 ml of normal saline over 15 min immediately before each CDDP administration to prevent CDDP-associated neurotoxicity, as indicated by our previous experience (Cascinu et al, 1995). Standard intravenous hydration was used: 2 h before initiation of the CDDP infusion, patients were hydrated with 1500 ml of 0.9% sodium chloride to which 20 mequiv. of potassium chloride and 15 mequiv. of magnesium sulphate were added. Post-hydration was continued for 2 h with 1000 ml of normal saline solution. As an antiemetic regimen, all patients received dexamethasone 20 mg in 50 ml saline given as an intravenous infusion over 15 min, 45 min before CDDP, and ondansetron 8 mg made up to 50 ml saline as an intravenous infusion over 15 min.

From the day before to the day after each chemotherapy administration, lenograstim was administered by subcutaneous injection at a dose of 5 μg kg$^{-1}$. One cycle of therapy consisted of eight weekly treatments. Patients who showed responsive or stable disease received a further 6 weeks of therapy. Full doses of anticancer drugs were given if the leucocyte count was 4000 μl$^{-1}$ and if the platelet count was greater than 100 000 μl$^{-1}$; when the leucocyte and platelet counts were less than this, we delayed the treatment by a week or until a complete recovery occurred. If grade 2 and 3 mucositis or diarrhoea occurred, treatment was delayed by a week or until normalization. For grade 4 toxicities, patients were removed from the study. No dose reductions were allowed.

### Evaluation of response and toxicity

Response and toxicity evaluation was based on the World Health Organization criteria (Miller et al, 1981), and on intention-to-treat analysis. Evaluation of response was performed after 8 and 14 weekly treatments, although toxicity was evaluated weekly. Overall incidence and intensity (Scott–Huskisson scale) of pain was recorded at baseline and after 8 weeks, as well as weight changes.

### Statistical methods

This was a multi-institutional phase II study; the primary objective was to determine the response rate and the toxicity of the weekly intensive chemotherapy. Secondary objectives were to measure the palliation of symptoms and survival.

According to the optimal two-stage phase II design, the treatment programme was designed to reject a response rate <30% (p0) and to provide a statistical power of 90% in assessing the activity of the regimen (in terms of response rate) as 45% (p1) (p1–p0 = 15%) for an alpha error less than 0.05 (Simon, 1989).

The 95% exact confidence interval (CI) for response was calculated. Survival time was calculated from the onset of chemotherapy. Chi-squared test with Yates correction and Wilcoxon test were used to assess the difference of pain between baseline and after eight chemotherapeutic treatments.

### RESULTS

Investigators from six institutions treated 22 advanced pancreatic cancer patients with this weekly intensive regimen. The study was

**Table 2** Toxicity (WHO criteria): number of episodes for each administration (week 1–8)

| Toxicity WHO grade | Week 1 1 or 2/3 or 4 | Week 2 1 or 2/3 or 4 | Week 3 1 or 2/3 or 4 | Week 4 1 or 2/3 or 4 | Week 5 1 or 2/3 or 4 | Week 6 1 or 2/3 or 4 | Week 7 1 or 2/3 or 4 | Week 8 1 or 2/3 or 4 |
|---|---|---|---|---|---|---|---|---|
| Leucopenia | 3/– | 4/– | 6/1 | 3/2 | 6/1 | 5/– | 2/1 | 4/1 |
| Thrombocytopenia | –/– | 5/– | 3/– | 4/– | 6/1 | 3/– | 5/1 | 4/– |
| Anaemia | 2/– | 3/– | 4/– | 3/1 | 5/– | 4/1 | 4/2 | 1/– |
| Mucositis | –/– | 1/1 | –/1 | 2/1 | –/– | 1/– | 2/– | 1/– |
| Diarrhoea | 1/– | 2/– | 2/1 | 1/– | 2/– | –/–\ | 1/– | 1/– |
| Nausea/vomiting | 5/2 | 7/2 | 7/1 | 4/1 | 6/1 | 2/1 | 1/1 | 2/1 |
| Neurotoxicity | –/– | –/– | –/– | 1/– | –/– | 2/– | –/– | –/– |

 © Cancer Research Campaign 1999

**Table 3** Toxicity (WHO criteria): number of episodes for each administration (week 9–14)

| Toxicity WHO grade | Week 9 1 or 2/3 or 4 | Week 10 1 or 2/3 or 4 | Week 11 1 or 2/3 or 4 | Week 12 1 or 2/3 or 4 | Week 13 1 or 2/3 or 4 | Week 14 1 or 2/3 or 4 |
|---|---|---|---|---|---|---|
| Leucopenia | 1/– | 3/1 | 4/1 | 1/1 | 3/– | 1/– |
| Thrombocytopenia | 1/– | 4/1 | 3/– | 2/– | 1/– | 3/– |
| Anaemia | 4/1 | 3/– | 4/– | 3/1 | 2/1 | 3/– |
| Mucositis | 2/1 | –/– | –/1 | 1/– | –/– | –/– |
| Diarrhoea | 1/– | –/– | 2/– | –/– | –/– | –/– |
| Nausea/vomiting | 2/1 | 1/– | 1/– | 1/– | 1/– | 1/– |
| Neurotoxicity | –/– | –/– | –/– | 1/– | –/– | 2/– |

closed earlier because it was evident that the nine objective responses, requested by the first stage of study, could not be achieved even completing the enrolment of all the planned 27 patients. The median follow-up from the start of treatment was 18 months (range 11–26 months). The characteristics of treated patients are detailed in Table 1.

### Tumour response

All patients had measurable disease on computerized tomography (CT) scan. Objective tumour response was seen in 3 out of 22 patients (13%, 95% CI 0–26%) with five patients showing stable disease and 14 progressing on therapy. All responses were obtained after the first 8 weeks.

### Patient survival

The median survival time of all 22 patients was 6 months (range 3–15 months), with a 1-year survival rate of 13%.

### Symptomatic effects

At baseline, pain was present in 15 out of 22 patients (68%), with a mean Scott–Huskisson scale value of 27.6 ± 23.8. After 8 weeks, pain was present in 9 out of 22 patients (40%) with a mean Scott–Huskisson scale value of 12.3±18.4. The difference between these mean values was statistically significant ($P = 0.022$). Eight patients (36%) (three partial responses and five stable disease) had a positive weight change after 8 weeks.

### Toxicity

Eight patients did not complete the first 8 weeks of treatment: three because of progressive disease and five because of toxicity (neutropenia and/or thrombocytopenia). No patient received the planned eight weekly treatments without some delay: in two patients, it was for a week; in three, for 2 weeks; in three, for 3 weeks; in three, for 4 weeks; and in one, for 8 weeks.

Of the eight patients who received six further weekly treatments, two did not complete the programme: one for progressive disease and one for toxicity. Some delay in weekly treatment was present in all the remaining six patients: in one patient, therapy was delayed for a week; in one, for 2 weeks; in one for 3 weeks; in two for 5 weeks; and in one for 8 weeks.

We did not observe any treatment-related death. Specific treatment-related toxicities per cycle administered are detailed in Tables 2 and 3. There was no evidence of cumulative toxicity in the following treatment weeks. The main common side-effect was leucopenia. Non-haematological toxicities were uncommon and mild.

### DISCUSSION

In advanced pancreatic cancer patients, the median survival is no longer than 3–4 months, a figure which has not been significantly influenced by chemotherapy during the last years (Ahlgren, 1996). Nevertheless, recent studies have shown that chemotherapy can improve overall survival compared with no treatment, without impairing quality of life (André et al, 1996; Glimelius et al, 1996; Rothenberg et al, 1996; Burris et al, 1997). Consequently, attempts to devise new chemotherapeutic regimens, with the aim of improving response rates and survival, are justified. We assessed in advanced pancreatic cancer the activity of a weekly regimen of CDDP, epi-ADR, LV and 5-FU that has been shown effective in advanced gastric cancer (Cascinu et al, 1997). The obtained response rate (13%) as well as the median survival time of 6 months are not substantially better than those achieved with older regimens such as FAM, FMS or 5-FU alone (Oster et al, 1986; Schnall and Macdonald, 1996). Furthermore, our results are similar to those obtained by Glimelius et al (1996) in the treatment arm of the randomized trial compared with best supportive care, but with a less toxic and expensive regimen.

Recently, comparable disappointing results were reported with another intensive regimen particularly active in advanced gastric cancer (Webb et al, 1997). ECF (epirubicin, cisplatin, 5-fluorouracil), in fact, showed a response rate of 17%, with a considerable toxicity that discourages its indiscriminate use highlighting the need for a careful selection of patients (Evans et al, 1996).

In pancreatic cancer, the notoriously difficult determination of objective response and the poor clinical conditions of several patients may account for the modest results achievable even by using intensive chemotherapeutic regimens. In fact, in pancreatic tumours, there is an important desmoplastic reaction induced by the tumour including inflammation and fibrosis within and around the tumour. Because this tissue does not necessarily shrink after chemotherapy, the size of the tumour on a CT scan may not reflect the true proportion of tumour response. Thus, the use of locally advanced pancreatic cancer as the sole indicator of response may yield misleading results (Ahlgren, 1996). In contrast, although metastatic lesions contain less desmoplastic component, advanced disease is associated with poor performance status and/or complications which can limit patient tolerance for chemotherapy, especially for aggressive regimens (Casper and Kelsen, 1995).

In our study, a favourable aspect was the presence of symptomatic effects, particularly a reduction in pain. After eight weekly

*© Cancer Research Campaign 1999* 

treatments, pain was present in 40% of patients compared with 68% at baseline, with a significant reduction in intensity as expressed by Scott–Huskisson scale values. However, in other clinical trials using less aggressive regimens, chemotherapy was found to improve quality of life and performance status (André et al, 1996; Glimelius et al, 1996). In particular, gemcitabine showed a very favourable toxicity profile and has demonstrated activity in this disease. Response rates ranged from 10% to 15% and the treatment reduced symptoms caused by the cancer in 20–30% of patients (Carmichael et al, 1995; Moore, 1996). A randomized trial comparing gemcitabine with 5-FU showed a significant improvement in clinical benefit (24% compared with 5%), as well as a significantly longer survival in patients receiving gemcitabine (Burris et al, 1997).

As in our case, subjective improvements observed in clinical trials were greater than expected from the tumour objective response rates. This can be due to the difficulty in properly quantifying objective response in this tumour, as discussed above. This being the case, the assessment of clinical benefit represents a new field of investigation in evaluating the activity and the role of chemotherapy in pancreatic cancer (Rothenberg et al, 1996). There is general acceptance that this new treatment end point needs to be explored in addition to classical end points (objective response and survival) in future chemotherapy trials in this disease.

In conclusion, the outcome of our intensive chemotherapy regimen does not support its use in advanced pancreatic cancer. Other drugs or regimens, achieving similar clinical benefit and survival, seem to be more convenient in terms of both economic aspects and patient's compliance.

### REFERENCES

Ahlgren JD (1996) Chemotherapy for pancreatic cancer. *Cancer* **78**: 654–663

André T, Lotz JP, Bouleuc C, Azzoust K, Houry S, Hannoun L, See J, Steso A, Avenin D and Izrael V (1996) Phase II trial of 5-fluorouracil, leucovorin and cisplatin for treatment of advanced pancreatic adenocarcinoma. *Ann Oncol* **7**: 173–178

Burris MA, Moore MJ, Andersen J, Green MR, Rothenberg ML, Modiano MR, Cripps MC, Portenoy RK, Storniolo AM, Tarassoff P, Nelson R, Dorr FA, Stephens CD and Von Hoff DD (1997) Improvement in survival and clinical benefit with gemcitabine as first-line therapy for patients with advanced pancreatic cancer: a randomized trial. *J Clin Oncol* **15**: 2403–2413

Carmichael J, Fink U, Russel RCG, Spittle MF, Harris A and Spiessl G (1995) Phase II study of gemcitabine in patients with advanced pancreatic cancer. *Br J Cancer* **73**: 101–105

Cascinu S, Cordella L, Del Ferro E, Fronzoni M and Catalano G (1995) Neuroprotective effect of reduced glutathione on cisplatin-based chemotherapy in advanced gastric cancer: a randomized double-blind placebo-controlled trial. *J Clin Oncol* **13**: 26–32

Cascinu S, Labianca R, Alessandroni P, Marcellini M, Silva RR, Pancera G, Testa E, Martignoni G, Barni S, Frontini L, Zaniboni A, Luporini G, Cellerino R and Catalano G (1997) Intensive weekly chemotherapy for advanced gastric cancer using 5-fluorouracil, cisplatin, epidoxorubicin, 6S-leucovorin, glutathione and filgrastim. A report from the Italian Group for the Study of Digestive Tract Cancer (GISCAD). *J Clin Oncol* **5**: 3313–3319

Casper ES and Kelsen DP (1995) Adenocarcinoma of the pancreas: overview of workup and management. *Adv Oncol* **11**: 17–22

Cocconi G, Bella M, Zironi S, Algeri R, Di Costanzo F, De Lisi G, Luppi G, Mazzocchi B, Rodino' C, Soldani M, Gilli G and Finardi C (1994) Fluorouracil, doxorubicin and mitomycin combination versus PELF chemotherapy in advanced gastric cancer: a prospective randomized trial of the Italian Oncology Group for Clinical Research. *J Clin Oncol* **12**: 2687–2693

Evans TRJ, Lofts FJ, Mansi JL, Gless JP, Dalgleish AG and Knight MJ (1996) A phase II study of continuous-infusion 5-fluorouracil with cisplatin and epirubicin in inoperable pancreatic cancer. *Br J Cancer* **73**: 1620–1624

Glimelius B, Hoffman K, Sjoden PO, Jacobsson G, Sellstrom H, Enander LK, Linne T and Svensson C (1996) Chemotherapy improves survival and quality of life in advanced pancreatic and biliary cancer. *Ann Oncol* **7**: 593–600

Kelly DM and Benjamin IS (1995) Pancreatic cancer. *Ann Oncol* **6**: 19–28

Lionetto R, Pugliese V, Bruzzi P and Rosso R (1995) No standard treatment is available for advanced pancreatic cancer. *Eur J Cancer* **31A**: 882–887

Moore M (1996) Activity of gemcitabine in patients with advanced pancreatic carcinoma. A review. *Cancer* **78**: 633–638

Miller AB, Hoodgstraten B, Staquet M and Winkler A (1981) Reporting results of cancer treatment. *Cancer* **47**: 207–214

Oster MW, Gray R, Panasci L and Perry MC (1986) Chemotherapy for advanced pancreatic cancer: a comparison of 5-fluorouracil, adriamycin, and mitomycin-c (FAM) with 5-fluorouracil, streptozotocin and mitomycin-c (FSM). *Cancer* **57**: 29–33

Palmer KR, Kerr M, Knowles G, Cull A, Carter DC and Leonard RC (1994) Chemotherapy prolongs survival in inoperable pancreatic cancer. *Br J Surg* **81**: 882–885

Rothenberg ML, Abruzzese JL, Moore MJ, Robertson JM and Wanebo HJ (1996) A rationale for expanding the end-points for clinical trials in advanced pancreatic carcinoma. *Cancer* **78**: 627–632

Schnall S and Macdonald JS (1996) Chemotherapy of adenocarcinoma of the pancreas. *Semin Oncol* **23**: 220–228

Simon R (1989) Optimal two-stage designs for phase II clinical trials. *Control Clin Trials* **10**: 1–10

Taylor I (1993) Should further studies of chemotherapy be carried out in pancreatic cancer? *Eur J Cancer* **29A**: 1076–1078

Webb A, Cunningham D, Scarffe JH, Harper P, Norman A, Joffe JK, Hughes M, Mansi J, Findlsy M, Hill A, Oates J, Nicolson M, Hickish T, O'Brien M, Iveson T, Watson M, Underhill C, Wardley A and Meehan M (1997) Randomized trial comparing epirubicin, cisplatin, and fluorouracil versus fluorouracil, doxorubicin and methotrexate in advanced oesophago-gastric cancer. *J Clin Oncol* **15**: 261–267

*© Cancer Research Campaign 1999*
